# A Pilgrim's Journey—When Parkinson's Disease Comes to an End in Nursing Homes

**DOI:** 10.3389/fneur.2018.01068

**Published:** 2018-12-11

**Authors:** Katharina Maria Lex, Philip Larkin, Jürgen Osterbrink, Stefan Lorenzl

**Affiliations:** ^1^Institute for Nursing Science and Practice, Paracelsus Medical University, Salzburg, Austria; ^2^UNIL - Université de Lausanne, CHUV - Centre hospitalier universitaire vaudois, Faculté de biologie et de médecine - FBM, Institut universitaire de formation et de recherche en soins - IUFRS, Lausanne, Switzerland; ^3^European Association of Palliative Care (EAPC), Milan, Italy; ^4^WHO Collaborating Centre for Nursing Research and Education, Salzburg, Austria; ^5^Department of Neurology and Palliative Medicine, Hospital Agatharied GmbH, Hausham, Germany; ^6^Department of Palliative Medicine, University Hospital of Munich, LMU, Munich, Germany

**Keywords:** morbus Parkinson, nursing homes, palliative care needs, ethnographic interview, assessments

## Abstract

Our interdisciplinary mixed-methods exploratory study was aimed at gaining empirical data on the medical and nursing demands of residents who are in a late stage of Parkinson Disease (PD) and are cared for in residential homes in Salzburg (Austria). In earlier studies it has been concluded that symptom burden of late stage PD patients is similar to or even higher compared with oncological patients. However, although all nine residents who took part in our study had severe limitations in performing their daily activities and experienced enormous restrictions in their mobility, they were quite content with their present living situations and did not show significant symptom burden. From the ethnographic family interviews that we conducted the following features emerged: a strong closeness in the family, an improved quality of life when the patients lived in the nursing home and fears about the future. Therefore, we concluded that living in a nursing home that provides for the needs of these patients is the best option for PD patients in the final stages of their disease as well as for their relatives.

## Introduction

Parkinson's Disease (PD) is the second most frequent neurodegenerative disease worldwide. It is estimated that between seven and 10 million people worldwide are living with PD (1). In Austria approximately 20 000 people live with PD (2). It has been estimated that the number of people who are older than 50 years and are diagnosed with PD will rise considerably from 4.1 to 4.6 million in 2005 to 8.7–9.3 million in 2030 (3). Bach et al. predicted that the number of people who will be affected by PD in 27 European countries, the US and Canada will increase by a factor of 1.6 between 2010 and 2035 (4). It is difficult to estimate the occurrence of PD in the population, as it varies considerably between different publications (5). Between one and two in 1,000 people are affected by PD (5). The average life expectancy of patients who are diagnosed with PD is around 15 years in Europe (6). In people who are 65 years or older the diagnosis of PD is a strong determining factor of long-term institutionalization, even when other chronic conditions and socio-demographic parameters are taken into account (7). Among male patients with PD 30% live in an institution and among female patients even 40 % (7).

The burden of symptoms of late stage PD patients has been described as similar or even higher than of those patients who suffer from oncological diseases (8). It has often been concluded that patients who suffer from advanced stages of PD have substantial unmet palliative care needs (8, 9). Families are often caring for their relatives over a long time period as well as round the clock with considerable personal, financial, social and health sacrifices (10). As the illness progresses and the abilities of the patients are increasingly reduced, the dependency on care rises significantly. At the same time health professionals often loose interest in patients and their families (9). Because medical options decrease and patients are not anymore eligible for pharmacological/medical studies.

### Objectives

In the nursing and residential homes in the city of Salzburg and the Salzburg county 4,384 people were being cared for in 2016 (11). Of these, 1,959 were 85 years of age or older (11). The exact number of residents being cared for in the nursing homes in the city of Salzburg and Salzburg county and being diagnosed with PD cannot be given, as there are no valid statistics.

Patients who are in an advanced stage of PD rarely participate in empirical studies (12). In Germany a non-representative study that analyzed death certificates from two different regions, demonstrated a tendency for more and more people dying in nursing homes over recent years: the number of people who died in nursing homes had risen from 12% in 2001 to 19% in 2011 (13). Only few empirical data are available for the care and medical situation of patients in an advanced phase of PD (14). In Salzburg and Salzburg county no data existed about the experience of residents with PD in their last phase of life, nor about their nursing and medical palliative care needs. The experiences of caring relatives are also unknown and their wishes when they are in close contact with their family members who suffer from severe PD and are cared for in a nursing homes. To get answers to these questions, the authors conducted this mixed-methods interdisciplinary, exploratory study.

## Methods

This study was approved by the Ethics Committee of the Salzburg county in November 2016 (415-E/2065/15-2016).

Residents were eligible to take part in our study if they matched our inclusion criteria. They had to be able to give written consent to their participation in the study or be able to instruct a legal attorney to give written consent on their behalf. This was the case for five participants. Another precondition for participation was the diagnosis of an advanced stage of PD (Hoehn and Yahr stage IV and V). At the time our visits and interviews took place, residents had to live in a nursing home either in Salzburg or in Salzburg county. The family member that was also interviewed in this study had to give written consent as well. Before we started with the first visit to a resident, the authors received a contract signed by the municipal authority of the city of Salzburg in which the city allowed the authors to conduct the study in the city's nursing homes. We conducted an exploarive, mixed-methods study.

### Instruments

We characterized each patient's situation using established scores. The Hoehn and Yahr scale and the Schwab and England Activities of Daily Living Scale were utilized to check each patient's inclusion criterion: being in an advanced stage of PD (Hoehn and Yahr stage 4 or higher). To assess the resident's severity of symptoms, the authors used the Unified Rating Scale for Parkinsonism (UPDRS). To describe the resident's quality of life the PDQ (Parkinson Disease Questionnaire) and the EQ-5D were used. To test if typical symptoms of the disease were present and to monitor their severity, the authors examined residents with the Edmonton Symptom Assessment System Parkinson Disease (ESAS-PD). To estimate the resident's satisfaction with medical and nursing support we used the patient satisfaction questionnaire short form (PSQ-18). The Charlson-Comorbidity Index Score was used to predict the resident's 10 year mortality. The Supportive and Palliative Care Indicator Tool (SPICT) was employed to detect the resident's palliative care needs and the necessity to develop individual care plans. The resident's family member's psychological situation was appraised by the Zarit Caregiver Burden Inventory (ZBI-22). To gain an insight into the quality of life of the resident's family member insofar as it relates to the experience of dementia, the researchers used the DEMQOL-Proxy-questionnaire.

Furthermore, we used the so-called “surprise question” (“Would you be surprised if this patient died within the next 6 months?”) as it is a simple tool which may help to judge estimates of the remaining life time. It is already part of clinical guidelines—e.g., the Gold Standard Framework in the UK. Originally it was developed to provide help with the decision about referral of patients to specialist palliative care treatment. It has been refined to help with the decision about the level of specialist palliative care treatment a patient might need. However, the accuracy of the surprise question, when used as a single assessment tool, varies considerably (15). Therefore, more scientific work is needed to clarify the prognostic accuracy of the surprise question (15). Hence, we intended to find out whether the surprise question can be a helpful tool in identifying patients who might profit from “active total care.” This term describes a combination of active treatment and, at the same time, the offer of medicine (and nursing care) which help -managing disabling symptoms and therefore make the time until death worth living (16). The results are shown in Table 1.

**Table 1:** Average and standard deviation of the assessment instruments.

	**Average**	**Standard deviation**	**Number of residents, who participated = *n***
PDQ	2.54	1.89	8
EQ5D	0.26	0.44	6
GESZ	51.55	40.78	6
ESASPD	1.67	2.65	6
LISK 1	0.89	0.93	5
LISK 2	0.78	0.67	5
PSQ 18	0.36	1.1	1
DEMQOLP	1.24	1.58	5
ZBI	0.26	0.86	2
UPDRS	1.77	1.66	9
Hoehn and Yahr	4.66	0.5	9
Schwab and England	2.66	1.41	9
CCI	0.1	0.70	3
Surprise question			9

The authors are aware of the difficulties in exactly predicting death, even when using the “surprise question” (17). Only in fewer than 4% of patients dying in the subsequent year the predicted mortality was above 80% when patients were admitted to the hospital a recent study showed (17).

The authors did intentionally not ask direct questions concerning advanced directives or end-of-life care as the local ethics committee was extremely worried about the study team asking direct questions on death and dying. The big fear was that the authors asking specific question might enlarge residents and family members' worries about their present living situation.

### Recruitment

**Figure 1:**
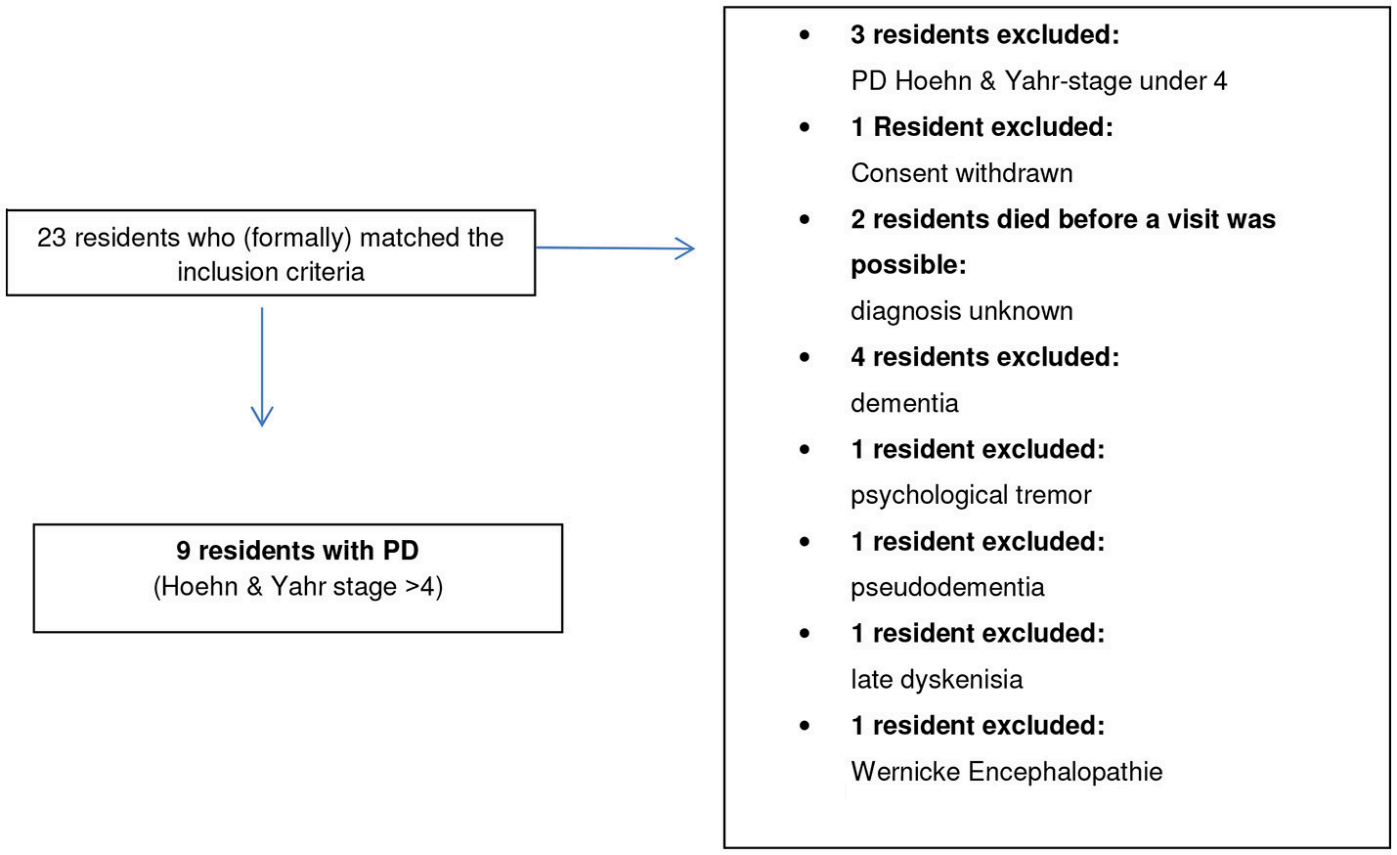
Recruitment.

Recruitment has been done in eight different nursing homes (four were located in the city of Salzburg and four in Salzburg county), see Table 2. Overall, 15 nursing homes with a total number of 1,478 residents are located in the city of Salzburg. Further 60 nursing homes (3,699 residents) are located in the Salzburg county (18). All nursing homes being either located in the City of Salzburg or in Salzburg county were contacted about the study either via telephone or by Mailing. The recruitment of the nursing home residents is shown in Figure 1. Recruitment has been supported by the chief doctor of the nursing homes in Salzburg who selected possible patients; we have distributed posters and flyers describing our study in the nursing homes. To gain extra attention (and possible study participation) of additional residents who were diagnosed with PD and who were not contacted by the chief doctor the flyers and posters were distributed. A message about the start of the study was announced via the electronic newsletter of the Institute for Nursing Research and Practice at the Paracelsus Medical University at Salzburg.

**Table 2:** Recruitment of the nursing home residents.

**Participating nursing home**	**Number of residents**	**Selected residents who met the inclusion criteria**	**Number of residents, who were seen by the research team**	**Number of residents in whom PD Hoehn and Yahr stage 4 or higher could be verified**
A (remote, rural area, Salzburg county, privately run)	121	8	8	2
B (remote, rural area, Salzburg county, run by the Austrian red cross)	52	2	2 (1 other resident died before the research team could visit the resident)	1
C (remote, rural area, Salzburg county, run by the local community)	36 (6 day care places)	1	0 (the resident died before the research team could visit the resident)	0
D (City of Salzburg, run by the City of Salzburg)	60	8	4 (4 residents family members were not reachable to ask for study consent/ did not want their resident to participate in the study)	3
E (City of Salzburg, run by the City of Salzburg)	96	6	3 (relatives of 2 residents were not reachable/did not give their consent to their residents participating in the study)	0
F (City of Salzburg, run by the City of Salzburg)	100	5	1 (relatives of 4 residents were not reachable/did not give their consent to their residents participating in the study)	0
G (remote, rural area, Salzburg county, run by the local community)	140	7	4 (2 proxies of attorneys needed more time to decide whether residents should participate in the study: in this time frame 2 residents died; 1 other family members did not give consent to participate in the study)	2
H (remote, rural area, Salzburg county, run by the “Salzburg Hilfswerk”)	66	1	1	1
	671	37	23	9

A total of 23 residents have been seen by the research team. Our gatekeepers were nursing home doctors, nursing home directors and people who are in charge of the organization of nursing homes in Salzburg and Salzburg county. Nursing home directors tested the criteria for inclusion into the study through their personal and professional nursing experiences and by checking the medical records of the residents that were available at the nursing home. The doctors who are in charge of the nursing homes at the city of Salzburg checked whether the residents who were willing to participate matched our inclusion criteria. The residents were visited in the nursing homes, where they lived by a professor for neurology and palliative care (SL) and by a nursing scientist who is also a nurse (KL). With all nine residents the assessment and the interview took place in the resident's room.

In the second phase of the visit (after completion of the various assessments) the authors invited relatives to participate in ethnographic interviews. This procedure was taken because we first had to confirm the diagnosis of idiopathic PD.

## Results

Of the 23 patients reported as idiopathic PD in only nine the diagnosis of PD with a Hoehn and Yahr stage 4 or higher could be verified (see Figure 1). Out of these nine patients it was possible to conduct family interviews in the style of ethnographic interviews in five cases.

The median age of the residents suffering from PD was 79.8 years. The socio-demographic data are shown in Table 3.

**Table 3:** Sociodemographic variables of patients.

	**Residents with PD**
Total number of residents (*n*)	9
Man (*n*)	4
Woman (*n*)	5
Age (range) (median)	59–94 (79.8)
Hoehn and Yahr stage 4	3
Hoehn and Yahr stage 5	6
Disease duration, years (range) (median)	6–20 (9)
Stay in residential home, years (range)	1–7
Specific anti PD-treatments e.g., deep brain stimulation in the past	1
Regular treatment by a neurologist	2

All residents had several comorbidities including polyneuropathy (*n* = 2), high blood pressure (*n* = 3), cerebrovascular diseases (*n* = 2), dementia (*n* = 3), cardiac insufficiency (*n* = 1), chronic lung disease (*n* = 1), gastritis (*n* = 2), spinal stenosis (*n* = 1), blindness (*n* = 1), type II diabetes (*n* = 1), and alcohol addiction (*n* = 1).

The averages and standard deviation of the assessment instruments we used can be seen in Table 1.

The research team was surprised by the fact that all residents included in the study were quite satisfied with their living situation, despite being severely impaired by their illness, especially in their overall autonomy. None of the residents had a feeding tube (PEG). Interestingly, one of them had had a PEG but due to intensive nursing care, he got rid of it and started to eat normally again. None of the residents had bothersome symptoms. All residents received a minimum dosage of anti-Parkinson medication (see Table 4). The illnesses of the not included residents can be seen in Table 5.

**Table 4:** Medication of participating residents.

**ID-number**	**Medication against PD**							**Pain medication**		**Anticonvulsive, antidepressive, and antipsychotic medication**						
	**Levodopa (mg)**	**Entacapone (mg)**	**Ropinirol (mg)**	**Pramipexol (mg)**	**Rasagilin (mg)**	**Biperi-dine (mg)**	**Rivas-tigmine (mg)**	**Paracetamol (mg)**	**Tramadol (mg)**	**Clonazepam (mg)**	**Levetiracetam (mg)**	**Mitrazapin (mg)**	**Lorazepan (mg)**	**Milnacipran (mg)**	**Quetiapin (mg)**	**Trazodon-hydrochlorid (mg)**
5	600			7,65				1,500	300	2	1,000					
9	300											15	1			
10	500				1									25	25	150
11			12			4	18									
18	250			2,62	1											
19	300	400													25	
22	300	200						500				15			50	

**Table 5:** Illnesses of the not included residents.

Dementia (not specified)	5
Pseudo dementia	1
Late dyskinesia	1
Psychological tremor	1
Wernicke encephalopathie	1
Morbus Parkinson Hoehn and Yahr stage 3	2
Morbus Parkinson Hoehn and Yahr stage 2	1
Unclassified	2

As a result of the surprise question, five nurses would be surprised if the residents would die within the next 6 months. Four nurses would not be surprised if the resident would die. All family members would be surprised if their relatives would die within the next 6 months.

We had to exclude 14 patients from our study for several reasons as shown in Figure 1. Surprisingly, the 10 residents, who did not match the PD diagnosis, had been treated with classical Parkinson medication. This has been in fact an ethically challenging result of our study. The authors reported this result to the residents, their family members and the nurses working in the residential homes. In four cases the doctor in charge was told about the result. In three cases the responsible doctor in the residential home was told. In several cases, SL gave some alternative treatment advice. Interfering in these cases is extremely difficult, as the researcher (SL) who could not verify the PD-diagnoses is not the doctor in charge, but acted in his role as a researcher.

### Qualitative Data

We conducted semi-structured, ethnographic, half-guided family interviews. We performed five interviews with daughters (2), husbands (1), sons (1), and (step-) brothers (1). One planned interview could not be conducted, although the relative (wife) was willing due to a severe speech impairment of the wife. The interviews were recorded and in the following paraphrased. The following features emerged from the interview data

- Strong closeness in the family
- Improved quality of life by living in the nursing home
- Fear about the future
- Feeling of responsibility for the resident, although s/he is being cared for in a nursing home

A positive aspect which emerged through the assessments and the interviews was a remarkable sense of closeness: family members had the feeling of symbiotically belonging to the resident and having the role of advocates, in the sense of caring and protecting the resident's needs who is vulnerable. A resident's half-brother told us about his biggest concern: “I worry whether I visit her enough.” Before the interview took place, he told the team that he visits his half-sister who is wheelchair bound and whose reactions and supposed understanding of verbal communication are extremely reduced, every other day.

In another interview situation the husband and his wife seemed to be very much one single person: the wife was enormously reduced in her physical and psychological expression, while the husband was extremely protective and very aware of his role as his “wife's advocate.”

The improved quality of life that both parties enjoy when the patient relocated into a nursing home can be illustrated by the following interview quotes. In contrast to any burdens when the patient moved into the nursing home, his/her new living and caring situation in the nursing home has even some beneficial aspects as well. It emerged from the interviews that the main reasons for nursing home treatment were frequent falls at home. Sometimes these falls had severe physical consequences: a resident's wife told us about her husband: “At home, he always fell. One time my son and I could not pick him up. He was too heavy. We had to call the ambulance. At the fall, he lost a tooth…. At home he was on his own and felt lonely; I was still working part-time. I was so worried. Then we moved him to the nursing home. He is much better here. The nurses look after him and cope well with his diabetes. And furthermore, he has something to occupy himself. On this ward lives a lady who enjoys playing cards. So they play cards together. Every day. He enjoys himself.”

A son was interviewed about his father's situation. He is completely bedridden and just able to use the words “yes” and “no” seemingly living in his own world: “Father enjoys eating. I think that is the only activity he still enjoys. Nurses care for him extremely well, so there is no burden for me that he lives in a nursing home.” “If it were possible to take him in a wheelchair and take a stroll through the park, that would be something I would enjoy tremendously.”

A husband used a very colorful picture to illustrate his fears concerning their future: “It all changes so quickly.” When asked about his biggest wish he answered: “If my wife's health situation could only improve to the situation it was in 2012, when we celebrated our golden wedding anniversary together.”

Caring relatives have the impression that they continue to be “in charge of the elderly relative.” Other family members and friends did not keep in contact with the residents. One daughter described this situation as the family was divided. None of the other family members kept in contact or visited her father. That is why she is the only person in her large family who feels that she is in charge of her father's social support and wellbeing. “That the other family members do not care about father's wellbeing has led to rifts within the family. I do not understand why the other family members do not care.”

A son told us what annoyed him most was that he from a large family with four other siblings was the only family member who regularly visited his father and felt responsible, including dealing with his financial and legal affairs. He is his legal guardian.

Caring family members seemed to be in a conflicting situation: although they were informed about the actual medical situation and the fact that death was probably to be expected in the near future, all of them hoped that their frail relative might get better. None of the family members expressed the wish that the old and ill relative may have a “good death” and avoid disturbing symptoms as for example dyspnea, fear or pain in the dying phase. When asked about her most important wish, the daughter who told us about the family rifts answered: “When the good fairy comes, she should take Parkinson's Disease away. Without Parkinson's, father would only have the usual symptoms of old age and everything would be fine.”

All the wishes about which the caring relatives spoke with the team were optimistic regarding the resident's future. One impressive wish was expressed by a resident's half-brother whose sister had been blind in one eye for the past 5 years and who was bed-bound. She needed complete help in all daily activities (ADLs). When he was asked what he would wish for his half-sister, he answered: “My largest wish is for her eyesight to improve.”

A resident's (step-) brother said: “It is like being on a pilgrimage.” With this statement he illustrated the ups and downs his sister and he experienced while living with PD, but finally she had reached a state where she wandered to the final destination.

## Discussion

The subject of residents who suffer from late stage PD and are cared for in nursing homes is internationally under researched. Only few empirical data are available on residents with PD Hoehn and Yahr stage 4 or higher who are cared for in residential homes [e.g., (19)]. That is why general knowledge on medical and nursing palliative care demands in these patients is limited.

The most important finding of our research has been that although patients who are in a progressive state of PD and are severely disabled, did not seem to have significant physical or emotional burden. Whilst residents were not satisfied with their overall health situation, they were not desperate. Relatives were still emotionally closely connected with the patients and expressed hope and confidence about the progression of the disease and the overall situation of the patients. Participation in our study was not a strain for patients with PD or for their family members. In contrast, we gained the impression that residents and carers, family members as well as nurses, enjoyed being able to contribute to our research.

The residents who participated in our study had been ill on average for 9 years. This is comparable with an earlier study of patients in a community setting in the United Kingdom (UK) (8). However, the residents in our study had a lower quality of life (EQ5D = 0.26) compared with the patients who participated in the UK study (8). Although the objectively measured quality of life has been low in our study, the residents were content with their present living situation and seemed to have a much higher “subjectively” experienced quality of life which we could not measure with the instruments we were using. Although all residents in our study were severely limited in their mobility, as they were either bedridden or wheelchair-bound, they did not make a point of it. In another study by Veronese et al. the prevalence of residents experiencing severe mobility problems was 66.7%. Especially people suffering from neurodegenerative diseases have severe constraints on their daily life activities (9). As the illness progresses and mobility becomes even more limited, patients and their family members get used to these limitations (9). If patients are not able to move on their own they are severely affected in their activities and well-being (20). On the other side being bedridden might also be a survival strategy. By lying down patients may gather their strengths to do other things that may be more important to them (20). In this particular study the findings were comparable to our results. None of the 32 elderly, bedridden patients described their situation as a good one, but they did not appear to suffer from their severe mobility difficulties (20). In our study only one resident, who was assessed H&Y IV and was very aware of his deteriorating physical and cognitive abilities, expressed his unhappiness about his overall situation.

Interestingly, the relatives seemed to have got used to the residents' situations. When we asked them about their wishes concerning the future they articulated a general wish for the patients to “get better again.” The ability to move did not seem to be particularly important.

Only two relatives answered the Zarit Caregiver Burden Inventory (ZBI). The very low ZBI- average of 0.26 is due to the fact that the relatives' actual suffering is low because the patient is no longer cared for at home. It might also be an indicator that the nursing care quality in the residential homes in Salzburg and the Salzburg county is high. This result shows a clear contrast to the result of our earlier study where we have recently shown that the ZBI-average is high when PD patients in the advanced stages are cared for at home (14). We have not yet assessed the ZBI factor of nurses in residential homes who care for the PD patients.

The finding that being close to one's family plays an important role for nursing home residents as well as for family members is consistent with other empirical data (21). It has already been shown, how important it is for residents to have close relationships to family and friends. These relationships are the foundation for relational dignity which is an important part of residents' concepts of their dignity (21).

All interviewed family members were convinced that their family members would survive the next 6 months; at the same time relatives were aware of the palliative phase of their family members and seemed to know that they might die sometime in the near future. Relatives of nursing home residents with late stage PD seem to experience highly ambivalent emotions. On the one hand they are very aware of their relatives' health situation as the palliative care phase had already started or was imminent and on the other hand they have optimistic wishes for their relatives' future which are not associated with a wish for a “good death.”

An important result was the strong feelings of uncertainty about the future. Many relatives expressed these worries in the interviews. In a qualitative study that explored PD patient's palliative needs (22). The main theme was the strong feeling of uncertainty and worry about the future (22). In this aspect our interviews are consistent with prior results (22).

The observation that family members “who are in charge of the resident” felt abandoned by the other family members or former friends of the resident has already been made in another study (9). Interestingly, being abandoned applies also to professionals: e.g., neurologists who do not care anymore about the elderly patients deteriorating (9).

Several residents had been diagnosed with PD years ago. However, this diagnosis could not be verified by the PD specialist (SL). All these residents had been treated with classical anti-Parkinson medication.

An idea to take some tension from not being able to verify a former PD-diagnosis and the n being stuck in the difficult situation of unclearness which doctor to confront with the “wrong” diagnosis—often having been diagnosed by a trusted GP- using the social constructivism method might help (23).

The research literature strongly suggests that people in advanced phases of PD should be looked after by a neurologist (12). As residents are no longer mobile enough, to travel and seek diagnosis and treatment by a neurologist, outreach neurologist services are strongly advised (12). Treatment by a neurologist leads to improved survival, fewer PD-related hospitalizations, lower health-care costs and greater patient satisfaction. Residents are also healthier, therefore it is extremely advisable to enable more PD-patients who are cared for in nursing homes, to have the benefits of getting medical treatment by neurologists as long as possible (24).

It is known that PD patients who are treated by a neurologist have an additional 6 years of survival compared with patients, who are looked after by family doctors or geriatricians (25). It has also been investigated that nursing home residents cared for by a neurologist are in better general health: they have lower rates of dementia, hip fracture, congestive heart failure, diabetes, ischemic heart disease, and stroke/TIA (25). To draw a reverse conclusion: residents who are not medically treated by a neurologist have a higher risk of suffering from comorbidities and the probability of an earlier death.

Taking the global shortage of neurologists (especially of those being experts in movement disorders) into account, specially qualified nurses might be a solution in ensuring a good medical and nursing care of residents having to live in nursing homes (26, 27). In some countries (e.g., Sweden) especially qualified PD-nurses take over an expert position in continuing medical and nursing care throughout the disease trajectory and offering a high amount of professional competency (27). It might be possible to qualify nurses according to the Swedish model and let these nurses care medically—in specific aereas- for affected residents. With this model the care of PD-residents might improve as less residents with wrong PD-diagnosis might be medically treated as having PD.

The authors had the impression that a high proportion of the residents' present quality of life was due to the caring and responsible work of the nurses and other carers who work at the Salzburg residential homes. The researchers were deeply impressed by the dedication of the nurses and the positive atmosphere in the nursing homes. The nurses were calm, lively and devoted.

The most important result of our study was that good palliative care is based on considerate nursing care and on minor and timely medical supplementation. This result confirmed the result of the study by Masel et al. (28). One of the main results of this study was that attentiveness and symptom management are important for PD patients (28).

Furthermore, reliable and validated PD and palliative care assessment instruments could not be adequately used in patients' late stages. Therefore, it was not possible to use classical, advanced statistical tests for analyzing data.

Based on our results it seems to make sense to triangulate methods when exploring patients' needs who are in an advanced phase of PD. With the validated assessment instruments experienced neurologists are able to verify the patients' illness stage. If one needs a more in- depth view of the experiences of patients and their relatives, qualitative methodology is essential. Combining interviews and observations with established assessment tools will lead to even more insights into the situations of the residents.

## Author Contributions

KL: research project organization and execution, manuscript writing of the first draft; SL: research project conception and execution, statistical analysis design, manuscript review and critique; JO: funding; PL: manuscript review.

### Conflict of Interest Statement

The authors declare that the research was conducted in the absence of any commercial or financial relationships that could be construed as a potential conflict of interest.
